# Polygenic risk scores in epilepsy

**DOI:** 10.1515/medgen-2022-2146

**Published:** 2022-09-22

**Authors:** Henrike O. Heyne

**Affiliations:** Digital Health Center, Hasso Plattner Institute for Digital Engineering, University of Potsdam, Potsdam, Germany; Hasso Plattner Institute for Digital Health at Mount Sinai, Icahn School of Medicine at Mount Sinai, New York, NY, USA; Department of Genetics and Genomic Sciences, Icahn School of Medicine at Mount Sinai, New York, NY, USA; Finnish Institute for Molecular Medicine (FIMM), University of Helsinki, Helsinki, Finland; Program for Medical and Population Genetics, Broad Institute of MIT and Harvard, Cambridge, MA, USA

**Keywords:** epilepsy, genome-wide association study, complex disease, polygenic score, risk prediction

## Abstract

An epilepsy diagnosis has large consequences for an individual but is often difficult to make in clinical practice. Novel biomarkers are thus greatly needed. Here, we give an overview of how thousands of common genetic factors that increase the risk for epilepsy can be summarized as epilepsy polygenic risk scores (PRS). We discuss the current state of research on how epilepsy PRS can serve as a biomarker for the risk for epilepsy. The high heritability of common forms of epilepsy, particularly genetic generalized epilepsy, indicates a promising potential for epilepsy PRS in diagnosis and risk prediction. Small sample sizes and low ancestral diversity of current epilepsy genome-wide association studies show, however, a need for larger and more diverse studies before epilepsy PRS could be properly implemented in the clinic.

## Introduction

Genetic information is increasingly used in clinical practice, also in disease prevention [1]. Here, genetic variants with large Mendelian effect sizes, which are mostly rare, are easiest to interpret in a genetic counseling setting as they are accordingly characterized by a high cumulative lifetime risk for a specific disease. While genome-wide association studies (GWAS) have demonstrated translational impact through identification of disease mechanisms and discovery and evaluation of therapeutic targets, the effect of individual GWAS loci on disease is small. (This is due to selection not permitting genetic variants with large effects on disease at high population frequencies [6].) It is well established that common genetic variants with small effects on specific diseases can be combined as polygenic (risk) scores (PRS or PGS). For five common disorders, a recent study showed a 3- to 5-fold increased disease risk for patients with a high disease-specific PRS, similar to risk conferred by rare monogenic variants [2]. Evaluating the utility of PRS in clinical genetic diagnosis is an active research area and there are multiple examples, particularly in breast cancer [3], [4] (including the breast cancer consortium in Germany [5]) and cardiovascular disease [6], that demonstrate how they could be implemented in routine clinical practice [7].

## Main

### Common genetic variants largely contribute to common forms of epilepsy

**Figure 1:**
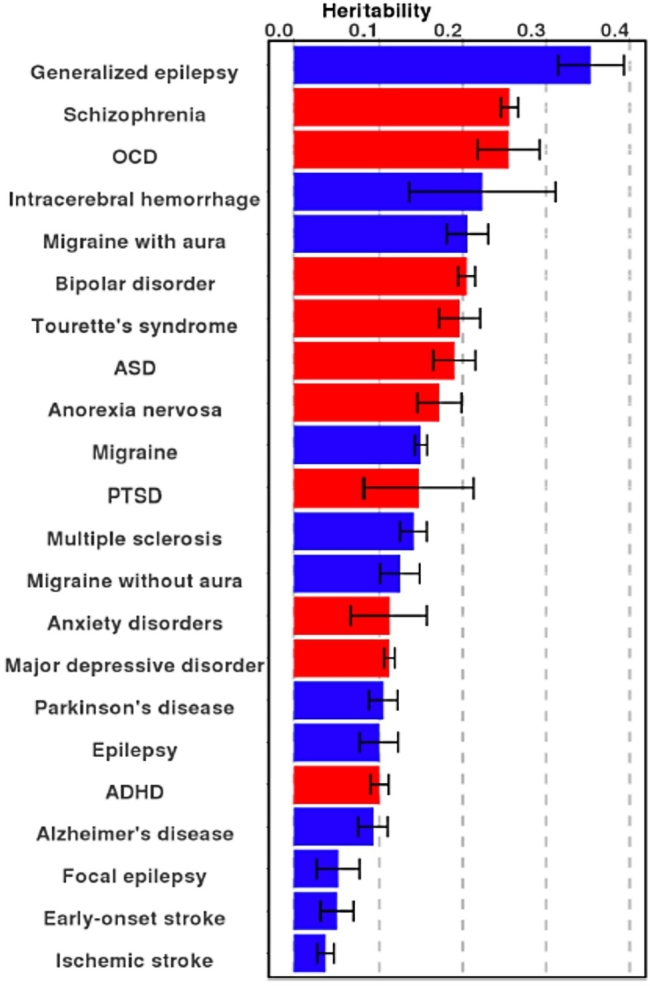
Adapted from [12]. Heritability estimates for different brain disorders. Red bars denote psychiatric disorders, while blue bars denote neurological disorders. ADHD – attention deficit hyperactivity disorder; ASD – autism spectrum disorder; ICH – intracerebral hemorrhage; OCD – obsessive-compulsive disorder; MDD – major depressive disorder; PTSD – post-traumatic stress disorder. Error bars show one standard error.

Epilepsy is a sometimes devastating neurological disorder characterized by unprovoked seizures, which affects approximately 1 % of individuals worldwide. Although epilepsy can be caused by acquired conditions such as stroke, tumor, or head injury, the majority of cases (ca. 70–80 %) are due to genetic influences [8]. While in about half of severe epilepsy cases single genetic mutations can be found as a cause [9], [10], GWAS have shown that common variants contribute particularly to milder and more common non-acquired forms of epilepsy [11]. These common epilepsy types are usually broadly summarized into genetic generalized epilepsy (here: generalized epilepsy) and non-acquired focal epilepsy (here: focal epilepsy). The proportion of heritability of generalized epilepsy attributed to common genetic variants with small individual effects, so-called single nucleotide polymorphism (SNP) heritability (or $h_{\mathrm{SNP}}^{2}$), is ca. 32 %, which is relatively high compared to other common brain disorders (see Figure 1, adapted from [12]). SNP heritability of focal epilepsy is lower, at about 9 %. Notably, there is a significant and substantial genetic correlation between subtypes of generalized epilepsy syndromes, suggesting a shared genetic basis for different generalized epilepsy types [11]. Generalized epilepsy subtypes show, however, no significant genetic correlation with focal epilepsy subtypes (with one exception that could, however, also arise from misclassification [11]). Interestingly, recent studies have shown a complementary contribution of family history and PRS to the risk of cancer [13] and other traits including epilepsy [14], thus emphasizing it is worth to consider both in disease prediction.

### Epilepsy GWAS have much smaller sample sizes than GWAS of other common diseases

**Figure 2:**
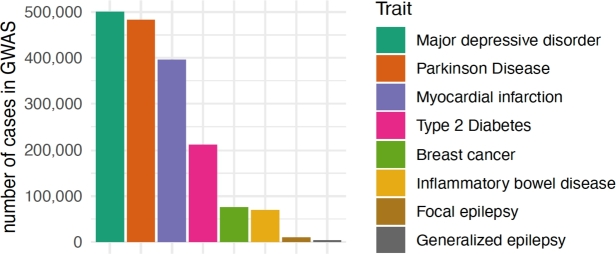
The number of cases in recent GWAS across different disease areas. Many common diseases have GWAS sample sizes of >100,000, while the largest GWAS in focal epilepsy (n=9,671) and generalized epilepsy (n=3,769) are substantially smaller at the time of writing.

Compared to other common complex diseases, specifically those where PRS are closest to getting implemented in clinical practice such as cardiovascular diseases [6] or breast cancer [4], [5], the sample size of the largest epilepsy GWAS is much smaller (see Figure 2). This also applies when comparing sample sizes of epilepsy GWAS to those of GWAS of diseases of similar prevalence such as inflammatory bowel disease or Parkinson’s disease (as of course larger sample sizes can be more easily achieved for traits with a higher population prevalence such as depression or cardiovascular diseases). As disease-specific PRS are calculated using data from the epilepsy discovery GWAS, using a small discovery GWAS potentially limits its clinical performance and thus utility. However, this is subject to change in the near future with an update of the International League Against Epilepsy (ILAE) Consortium on Complex Epilepsies currently underway.

It has been consistently shown across diseases that PRS predict disease risk up to several times more accurately in Europeans than in non-Europeans. This is a consequence of the substantial underrepresentation of non-European samples in most GWAS [15]. In the latest ILAE epilepsy GWAS the vast majority of samples came from individuals of European ancestry. The research could thus be more beneficial to individuals of European ancestry, who, however, often already receive better healthcare than other ancestry groups.

**Figure 3:**
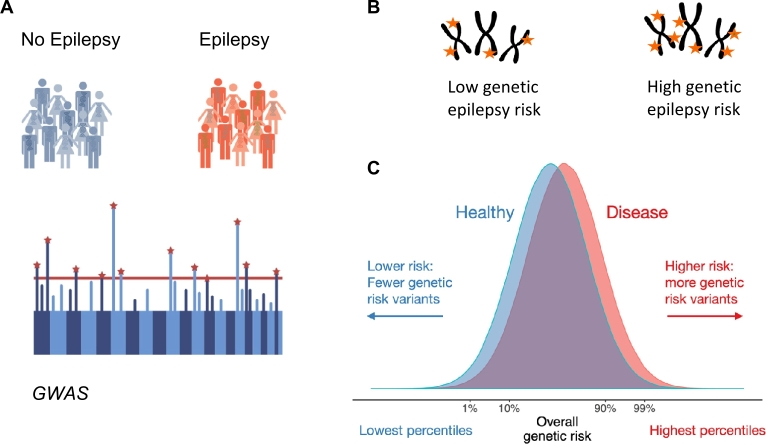
Calculation of epilepsy PRS. (A) First, genetic differences between the groups of individuals with and without epilepsy are identified in a genome-wide association study (GWAS). A GWAS finds genetic markers that decrease or increase the risk for epilepsy. **(B)** In a second independent target cohort, thousands of epilepsy risk or protective markers are then weighted and counted in each individual to obtain a single number representing the overall genetic liability for epilepsy: the epilepsy PRS. **(C)** On a group level, the epilepsy PRS (or genetic burden for epilepsy) can then be compared between epilepsy cases and controls in the target cohort.

### Elevated epilepsy PRS in epilepsy cases compared to controls

While individual genetic markers are significantly associated with a range of epilepsy phenotypes in GWAS their individual effect sizes are small. The predictive power and thus clinical utility of individual epilepsy GWAS loci is thus limited. However, recent years have seen a rapid advancement in the way how thousands of small-effect associations could be combined to a PRS that can have substantial effect sizes and thus potential clinical relevance (Figure 3). Analogously to other common diseases, recent studies found that individuals with epilepsy also had a significantly elevated epilepsy PRS compared to population-based controls [16], [17]. Here, the disease-specific PRS was particularly elevated in generalized epilepsy. There was only a modest polygenic burden in focal epilepsy, which is expected given its much lower SNP heritability than generalized epilepsy [11]. While there is a higher burden of ultrarare variants in generalized than in focal epilepsy, well-established Mendelian genes can only be found for focal epilepsy [18]. It is thus unclear how different rare variant burdens may contribute to the difference in focal and generalized epilepsies’ SNP heritability. An important limitation of both studies is that they were only conducted in individuals of European ancestry. Given the known limited transferability of PRS across continental ancestries [15], further studies in more diverse populations are needed to understand the clinical utility of epilepsy PRS derived from currently available GWAS in individuals of non-European ancestry. More importantly, however, as applies for other diseases, epilepsy GWAS need to include more individuals of non-European descent to potentially offer epilepsy risk prediction for any humans worldwide regardless of their ancestral background.

### Epilepsy PRS’ clinical potential as a marker for epilepsy risk

The absolute lifetime risk to develop generalized epilepsy is usually <0.1–0.5 %. Even in the tails of the generalized epilepsy PRS distribution the increased disease risk of individuals with a high genetic liability does not usually exceed 5× the risk of the population average. Therefore, the absolute lifetime risk for epilepsy would be approximately 1–2 % for individuals with a high genetic liability for epilepsy. This low absolute risk limits the clinical utility of epilepsy risk prediction in healthy individuals. However, this is different in individuals at high epilepsy risk. The clinical guidelines of the ILAE require at least one unprovoked seizure and at least a 60 % chance of a second seizure for an epilepsy diagnosis [19]. In clinical practice, making this diagnosis is often difficult and up to 25 % of epilepsy patients could initially be misdiagnosed [20]. Genetic information in the form of an epilepsy PRS has thus a great potential to serve in the future as a biomarker for epilepsy risk in predicting another seizure in individuals with one unspecified seizure event, of whom ca. 50 % eventually develop epilepsy [21], as these biomarkers are currently lacking. Recent preliminary results in >269,000 Finns from the FinnGen study indicate this may be possible [22]. Here, the authors are currently investigating the association between the epilepsy PRS and a later epilepsy diagnosis in participants who suffered seizures for which the cause was unclear.

## Summary and outlook

The high heritability of common forms of epilepsy, particularly genetic generalized epilepsy, indicates a great potential for common genetic markers to serve as a biomarker for epilepsy diagnosis and risk prediction. The quite distinct genetic basis of focal and generalized epilepsy also indicates a potential utility for PRS in helping to distinguish between epilepsy subtypes. The low sample sizes and low ancestral diversity of current epilepsy GWAS show, however, a great need for larger studies specifically including non-European individuals before epilepsy PRS could be properly implemented in the clinic. Due to the low lifetime prevalence of epilepsy, the clinical utility of PRS for epilepsy risk prediction would be modest in the average population, but PRS have great potential in clinical groups at high epilepsy risk, e. g., individuals with an unspecified seizure event, for whom such biomarkers are currently lacking.

## Glossary

ILAE (International League Against Epilepsy) – The world’s largest association of physicians and other health professionals in epilepsy, founded in 1909.

GWAS (genome-wide association study) – The study of genotype–phenotype associations for millions of genetic markers across the whole genome.

Heritability (h^2^) – The proportion of phenotypic variance that can be explained by common genetic markers, often estimated from GWAS.

SNP (single nucleotide polymorphism) – A single nucleotide at a specific position in the genome that is different in a large fraction (typically more than 5 % or 1 %) of individuals in a population. Most genetic markers in GWAS are SNPs.

PRS (polygenic risk score) – The sum of genetic risk markers equivalent to an individual’s genetic liability to a specific disease, calculated using GWAS data of an independent discovery cohort.

Discovery (or base) cohort – GWAS results containing genetic markers that increase/decrease the risk for a given disease.

Target cohort – The research cohort in which PRSs are calculated consisting of individual-level genotype and phenotype data.
